# Supplementary material for: Understanding the customer experience in human-computer interaction: a systematic literature review
Source: PeerJ Comput Sci. 2023 Feb 10;9:e1219. doi: 10.7717/peerj-cs.1219 (PMC10280563; doi:10.7717/peerj-cs.1219)
Supplement: Supplemental Information 1 [file peerj-cs-09-1219-s001.zip › Supplementary material No1 - CX definitions.docx]

## **Customer experience definitions.**

| **Nº** | **Author** | **Year** | **Domain** | **CX Definition** | **Articles that cite this definition** |
| --- | --- | --- | --- | --- | --- |
| 1 | B. Schmitt (Schmitt, 1999) | 1999 | Marketing (Experiential marketing) | “Experiential marketing focuses on customer experiences. Experiences occur because of encountering, undergoing, or living through things. Experiences provide sensory, emotional, cognitive, behavioral, and relational values that replace functional values”. | (Godovykh and Tasci, 2020), (Waqas *et al.*, 2020), (Verhoef *et al.*, 2009), (Gentile *et al.*, 2007), (“Phil” Klaus and Maklan, 2012), (Brakus *et al.*, 2009), (Walls, 2013), (Lee *et al.*, 2018), (Lemon and Verhoef, 2016), (Becker and Jaakkola, 2020) |
| 2 | C. Shaw and J. Ivens (Shaw and Ivens, 2002) | 2002 | General | “The customer experience is a blend of a company’s physical performance and the emotions evoked, intuitively measured against customer expectations across all moments of contact”. | (Klaus and Maklan, 2011) |
| 3 | C. Shaw (Shaw, 2004) | 2005 | General | “A customer experience is an interaction between an organization and a customer. It is a blend of an organization’s physical performance, the senses stimulated, and emotions evoked, each intuitively measured against customer expectations across all moments of contact”. | (Ren *et al.*, 2016) |
| 4 | O. A. Mascarenhas, R. Kesavan, and M. Bernacchi (Mascarenhas *et al.*, 2006) | 2006 | General | “Total customer experience is a totally positive, engaging, enduring, and socially fulfilling physical and emotional customer experience across all major levels of one’s consumption chain and one that is brought about by a distinct market offering that calls for active interaction between consumers and providers”. | (Deshwal and Bhuyan, 2018) |
| 5 | C. Meyer and A. Schwager (Meyer and Schwager, 2007) | 2007 | General | “Customer experience is the internal and subjective response customers have to any direct or indirect contact with a company. Direct contact generally occurs during purchase, use and service and is usually initiated by the customer. Indirect contact most often involves unplanned encounters with representations of a company's products, services, or brands and takes the form of word-of-mouth recommendations or criticisms, advertising, new reports, reviews, and so forth”. | (“Phil” Klaus and Maklan, 2012), (Lemon and Verhoef, 2016), (Lemke *et al.*, 2011), (Becker and Jaakkola, 2020), (Laming and Mason, 2014), (Khan *et al.*, 2020), (Klaus, 2015), (Bolton *et al.*, 2018), (Ren *et al.*, 2016), (Peng *et al.*, 2015), (Ali *et al.*, 2018), (Hoyer *et al.*, 2020), (Kim *et al.*, 2011), (Seppanen and Laukkanen, 2016), (Maslov, 2019), (Belabbes *et al.*, 2017), (Shin *et al.*, 2019), (Sujata *et al.*, 2016), (Rageh *et al.*, 2013), (Loureiro and Sarmento, 2017), (Rose *et al.*, 2012) |
| 6 | C. Gentile, N. Spiller, and G. Noci (Gentile *et al.*, 2007) | 2007 | General | “The customer experience (CX) concept is defined as an evolution of the concept of relationship between the company and the customer. The CX originates from a set of interactions between a customer and a product, a company, or part of its organization, which provoke a reaction. This experience is strictly personal and implies the customer’s involvement at different levels (rational, emotional, sensorial physical and spiritual). Its evaluation depends on the comparison between a customer’s expectations and the stimuli coming from the interaction with the company and its offering in correspondence of the different moments of contact or touchpoints”. | (“Phil” Klaus and Maklan, 2012), (Lemke *et al.*, 2011), (Bolton *et al.*, 2018), (Srivastava and Kaul, 2016), (Kim *et al.*, 2011), (Seppanen and Laukkanen, 2016), (Belabbes *et al.*, 2017), (Sathish and Ganesan, 2015), (Chauhan and Manhas, 2017), (Sujata *et al.*, 2016), (Boakye *et al.*, 2016) |
| 7 | J. W. Schouten, J. H. McAlexander, and H. F. Koenig (Schouten *et al.*, 2007) | 2007 | General | “Transcendent customer experiences (TCEs) are characterized by feelings such as self-transformation or awakening, separation from the mundane, and connectedness to larger phenomena outside the self. TCEs may also be marked by emotional intensity, epiphany, singularity and newness of experience, extreme enjoyment, oneness, ineffability, extreme focus of attention, and the testing of personal limits”. |  |
| 8 | R. Johnston, and G. Clark (Johnston and Clark, 2008) | 2008 | Management (Service operation management) | “The customer experience is the customer’s direct and personal interpretation of, and response to, their interaction with and participation in the service process, and its outputs, involving their journey through a series of touch points/steps”. |  |
| 9 | P. C. Verhoef, K. N. Lemon, A. Parasuraman, A. Roggeveen, M. Tsiros, and L. A. Schlesinger (Verhoef *et al.*, 2009) | 2009 | Retail | “The customer experience construct is holistic in nature and involves the customer’s cognitive, affective, emotional, social, and physical responses to the retailer. This experience is created not only by those elements which the retailer can control (e.g., service interface, retail atmosphere, assortment, price), but also by elements that are outside of the retailer’s control (e.g., influence of others, purpose of shopping). Additionally, the customer experience encompasses the total experience, including the search, purchase, consumption, and after-sale phases of the experience, and may involve multiple retail channels”. | (Lemon and Verhoef, 2016), (Lemke *et al.*, 2011), (Grewal *et al.*, 2009), (Becker and Jaakkola, 2020), (Klaus, 2015), (Bolton *et al.*, 2018), (Srivastava and Kaul, 2016), (Kim *et al.*, 2011), (Belabbes *et al.*, 2017), (Roy *et al.*, 2020), (Salehi *et al.*, 2013), (Singh, 2019) |
| 10 | K. Ghose (Ghose, 2009) | 2009 | Brand | “Customer experience is defined as the user’s interpretation of his or her total interaction with the brand”. | (Shin *et al.*, 2019) |
| 11 | R. Gopalan and B. Narayan (Gopalan and Narayan, 2010) | 2010 | Tourism | “The customer experience in tourism consists of an assorted bundle of experiences, starting with the immigration desk and customs clearance at the airport”. |  |
| 12 | C. Shaw, Q. Dibeehi, and S. Walden (Shaw *et al.*, 2010) | 2010 | General | “A customer experience is an interaction between an organization and a customer as perceived through a customer’s conscious and subconscious mind. It is a blend of an organization’s rational performance, the senses stimulated, and emotions evoked, and intuitively measured against customer expectations across all moments of contact”. |  |
| 13 | H. Yang, T. Yang, T. and Wen (Yang *et al.*, 2010) | 2010 | General | “The customer experience is defined as an interaction between an organization and a customer. It is a blend of an organization’s physical performance, the senses stimulated, and emotions evoked, each intuitively measured against customer expectations across all moments of contact”. |  |
| 14 | U. Walter, B. Edvardsson, and A. Öström (Walter *et al.*, 2010) | 2010 | Restaurant | “Customer experience is defined as the customer’s direct and indirect experience of the service process, the organization, the facilities and how the customer interacts with the service firm’s representatives and other customers”. “These in turn create the customer’s cognitive, emotional, and behavioral responses and leave the customer with memories about the experience. Further, a customer experience is seen as occurring in a commercial context and is therefore regarded as being shaped and offered by a service company, which has a commercial purpose”. |  |
| 15 | F. Lemke, M. Clark, and H Wilson (Lemke *et al.*, 2011) | 2011 | Business (B2B and B2C) | “The customer experience is the customer’s subjective response to the holistic direct and indirect encounter with the firm, including but not necessarily limited to the communication encounter, the service encounter, and the consumption encounter”. | (“Phil” Klaus and Maklan, 2012), (Kumar and Anjaly, 2017), (Belabbes and Oubrich, 2018), (Buttle and Maklan, 2015) |
| 16 | H. Yu Hsu, and Hung-Tai Tsou (Hsu and Tsou, 2011) | 2011 | Blog (Online blog environments) | “Customer experiences of blogs can be viewed as consumers’ emotional responses to environmental psychology while they are spurred by the environmental cues”. |  |
| 17 | P. Klaus and S. Maklan (Klaus and Maklan, 2011) | 2011 | Sport (Mountain-biking extreme sport camp) | “Customer experience is the customers comprehensive assessment of social interaction, personal hedonic benefits, destination attributes, their relationship to the environment (social and nature) and their personal growth related to challenges and sense of communities”. |  |
| 18 | X. Zhang, G. Bu, S. Wu, and Q. Xie (Zhang *et al.*, 2011) | 2011 | Automobile | “Customer experience is the degree of customers’ perception, experience and recognition of the goods and services, involving human’s sensibility factors, such as senses, intuition, mood, emotion and rational factors, such as intelligence, thinking, and can reflect the comprehensive evaluation of products or services from consumers, and reflect products or services on the consumer's value”. |  |
| 19 | C. Zhang, Z. Wen-An, C. Jian, and G. Hai-Sheng (Chen *et al.*, 2012) | 2012 | Telecommunication (Telecommunication services) | “Customer experience is the result of the sum of observations, perceptions, thoughts, and feelings arising from interactions and relationships (direct and indirect) over an interval of time between a customer and their provider(s)”. |  |
| 20 | J. Sirapracha and G. Tocquer (Sirapracha and Tocquer, 2012) | 2012 | Telecommunication  (Telecommunication industry) | “Customer experience is defined as the outcome of customers’ interactions with the firm, including the interaction with the staff, self-service technologies, and the service environment”. | (Roy *et al.*, 2020), (Chahal and Dutta, 2015) |
| 21 | P. Klaus and S. Maklan (“Phil” Klaus and Maklan, 2012) (Klaus and Maklan, 2013) | 2012, 2013 | Bank (Retail banking)  Mortgage  Fuel and service station  Retail (Luxury retailing) | “The customer experience is the customer’s cognitive and affective assessment of all direct and indirect encounters with the firm relating to their purchasing behavior”. |  |
| 22 | S. Bagdare and R. Jain (Bagdare and Jain, 2013) | 2013 | Retail | “The customer experience is the sum total of cognitive, emotional, sensorial, and behavioral responses produced during the entire buying process, involving an integrated series of interaction with people, objects, processes, and environment in retailing”. |  |
| 23 | P. Klaus (Klaus, 2013) | 2013 | E-Commerce (Amazon) | “Online customer service experience (OCSE) is the customers’ mental perception of interactions with a company’s value proposition online. These mental perceptions in turn drive a set of outcomes, namely benefits, emotions, judgments (including perceived value) and intentions”. “OCSE is the customers’ overall mental perception of their interaction with the online service provider and other customers expressed in its dimension’s functionality and psychological factors. The overall OCSE mental perception and its relationship to the customers’ value perception drive their subsequent purchasing and word-of-mouth behavior”. | (Singh, 2019) |
| 24 | M. Watkinson (Watkinson, 2013) | 2013 | General | “The customer experience is the qualitative aspect of any interaction that an individual has with a business, its products, or services, at any point in time”. |  |
| 25 | S. Joshi (Joshi, 2014) | 2014 | Telecommunica-tion (Cellular mobile devices) | “Customer experience is defined as the sum of all experiences that a customer has at every touchpoint of the customer-company relationship”. |  |
| 26 | C. Laming and K. Mason (Laming and Mason, 2014) | 2014 | Airline | “Customer experience may be defined as the physical and emotional experiences occurring through the interactions with the product and/or service offering of a brand from point of first direct, conscious contact, through the total journey to the post-consumption stage”. |  |
| 27 | J. Zhang (Zhang, 2014) | 2014 | General | “Customer experience is defined as perception and emotional reaction during the process of performing an act or experiencing an event. To be further, it is the perception and emotional reaction generated by customers during the interaction with particular product or service, individuals, or organizations, when they try to satisfy their requirements during events”. |  |
| 28 | A. De Keyser, K. N. Lemon, P. Klaus, and T. L. Keiningham (De Keyser *et al.*, 2015) | 2015 | General | “Customer experience is comprised of the cognitive, emotional, physical, sensorial, and social elements that mark the customer’s direct or indirect interaction with a (set of) market actor(s)”. | (Lemon and Verhoef, 2016), (Roy *et al.*, 2020), (Klaus, 2018) |
| 29 | P. Klaus (Klaus, 2015) | 2015 | General | “The customer experience definition ranges from a customer’s actual and anticipated purchase and consumption experience, a distinctive economic offering or the result of encountering, undergoing, or living through things, to the notion of the new, experience-seeking consumer as co-creator of value and experience. It includes pre- and post-service encounter/purchase/consumption experiences, addresses emotional as well as functional dimensions of quality, and includes the customer’s social context”. “The customer experience is the customer’s cognitive and affective assessment of all direct and indirect encounters with the firm relating to their purchasing behavior”. |  |
| 30 | J. Peng, X. Zhao, and A. S. Mattila (Peng *et al.*, 2015) | 2015 | Hotel (Budget hotel) | “The customer experience is defined as guests’ emotional evaluations of their consumption episode”. |  |
| 31 | Y. Pei, W. Xue, D. Li, J. Chang, and Y. Su (Pei, Xue, Li, *et al.*, 2015) (Pei, Xue, Su, *et al.*, 2015) | 2015 | E-Commerce (B2C e-commerce enterprises) | “E-Commerce customer experience is the process in which customers obtain the special feeling, form cognition and evaluation, thus affect the meeting of psychological demands and decision making through a set of shopping conditions and environments and interactive service that E-Commerce websites and operators provide. Customer experience becomes an important factor affecting consumers’ online shopping behaviors, and good customer experience can promote consumers’ web-based purchase behaviors”. |  |
| 32 | S. Chen and C. Lin (Chen and Lin, 2015) | 2015 | Blog | “Customer experience is the degree of sensory, emotional, and cognitive impacts generated by users when participating in and observing blogs, which elevates their interest, motivations, and recognition, and subsequently adds value to the blogs”. |  |
| 33 | M. Seppänen and I. Laukkanen (Seppanen and Laukkanen, 2016) | 2015 | Business (Business models) | “Customer experience emerges from interactions between a firm and its customer, as perceived through a customer’s conscious and subconscious mind; it is not just about the ‘what’ but also about the ‘how’”. |  |
| 34 | F. Buttle and S. Maklan (Buttle and Maklan, 2015) | 2015 | Management | “Customer experience is the cognitive and affective outcome of the customer’s exposure to, or interaction with, a company’s people, processes, technologies, products, services, and other outputs”. |  |
| 35 | S. Chang and R. Lin (Chang and Lin, 2015) | 2015 | General | “Total customer experience refers to customer satisfaction of material and emotional needs during all principal processes in the personal consumption chain”. |  |
| 36 | D. Menachem, S. Joshi, S. Bhatia, A. Roy, and J. Saini (Menachem *et al.*, 2015) | 2015 | Telecommunica-tion (Telecom operators) | “The customer experience involves the total participation during the customer lifecycle, including the search, consumption, purchase and after-sale phases of the experience, measured against the customer expectations which will eventually augment the customer retention and loyalty conclusively affecting the business value”. |  |
| 37 | K. G. Boakye, C. Chiang, and X. Tang (Boakye *et al.*, 2016) | 2016 | Market (Electronic product smartphone) | “The customer experience is a psychological state in which consumers engage in cognitive and affective processing of the product’s usage”. |  |
| 38 | K. N. Lemon and P. C. Verhoef (Lemon and Verhoef, 2016) | 2016 | Marketing | “Customer experience is a multidimensional construct focusing on a customer’s cognitive, emotional, behavioral, sensorial, and social responses to a firm’s offerings during the customer’s entire purchase journey”. | (Becker and Jaakkola, 2020), (Khan *et al.*, 2020), (Bolton *et al.*, 2018), (Zaharia and Schmitz, 2020) |
| 39 | K. N. Lemon and P. C. Verhoef (Lemon and Verhoef, 2016) | 2016 | Marketing | “Customer experience is the customer’s “journey” with a firm over time during the purchase cycle across multiple touchpoints”. | (Hoyer *et al.*, 2020) |
| 40 | C. Shaw and R. Hamilton (Shaw and Hamilton, 2016) | 2016 | General | “A customer experience is a customer’s perception of their rational, physical, emotional, subconscious, and psychological interaction with any part of an organization. These perceptions inﬂuence customer behaviors and build memories, which drive customer loyalty and thereby aﬀect the economic value an organization generates”. |  |
| 41 | M. Lipkin (Lipkin, 2016) | 2016 | General | “Customer experience formation (CXF) es defined as the different ways in which an experience is realized through intermediation between individual and the context, as well as is shaped from a more actor-related and often abstract, contextual viewpoint”. |  |
| 42 | S. K. Roy, M. S. Balaji, S. Sadeque, B. Nguyen, and T. C. Melewar (Roy *et al.*, 2017) | 2017 | Retail (Smart retail technology) | “Smart customer experience (SCE) is defined as a component of smart retailing which focuses specifically on the technology-mediated (e.g. connected technology like Internet of Things) retailing experiences”. |  |
| 43 | M. Yoshida (Yoshida, 2017) | 2017 | Sport | “Sport consumer experience is defined as sport consumers’ cognitive, affective, social, and physical reactions to direct (e.g., purchase and consumption) and indirect (e.g., media and social network) encounters with a sport organization, its products, and other consumers”. | (De Keyser *et al.*, 2020) |
| 44 | L. Boureanu (Boureanu, 2017) | 2017 | General | “Customer experience is the sum total of interactions with the customer—from noticing a company, a product, or a service via purchasing it to using the product or service”. |  |
| 45 | R. Jain, J. Aagja, and S. Bagdare (Jain *et al.*, 2017) | 2017 | General | “Customer experience is the aggregate of feelings, perceptions and attitudes formed during the entire process of decision making and consumption chain involving an integrated series of interaction with people, objects, processes, and environment, leading to cognitive, emotional, sensorial, and behavioral responses”. |  |
| 46 | B. Larivière, D. Bowen, T. W. Andreassen, W. Kunz, N. J. Sirianni, C. Voss, N. V. Wünderlich, and A. De Keyser (Larivière *et al.*, 2017) | 2017 | Service encounter | “The customer experience is the totality of cognitive, emotional, behavioral, sensorial, and social responses that result from interactions with other parties (e.g., customers, and technology)”. |  |
| 47 | J. Liu, G. Z. Hu, Y. Yu, W. J. Yi, and L. L. Zuo (Liu *et al.*, 2017) | 2017 | E-Commerce (Recommenda-tion system) | “Customer experience is reflection and feeling during the process of interaction with enterprises under the situation of human-computer interaction”. |  |
| 48 | C. Homburg, D. Jozić, and C. Kuehnl (Homburg *et al.*, 2017) | 2017 | Management | “Customer experience is the evolvement of a person’s sensorial, affective, cognitive, relational, and behavioral responses to a firm or brand by living through a journey of touchpoints along prepurchase, purchase, and post purchase situations and continually judging this journey against response thresholds of co-occurring experiences in a person’s related environment”. |  |
| 49 | N. Tran Thi Tuyet and Y. Hara (Tran *et al.*, 2017) | 2017 | Retail (Traditional (wet market) and modern (supermarket) | “Customer experience is defined as an evolution of the concept of relationship between the service provider (retailer) and the customer and put it in the customer’s point of contact in service such as contact with product, store atmosphere, other customers, retailers, and context”. |  |
| 50 | P. Klaus (Klaus, 2018) | 2018 | Healthcare (Patients) | “The customer experience as "patient experience" is a service provision in an environment where the goals of the customer can be complex, and where appropriate service to the customer may take the provider beyond the typical customer service approach of striving to provide immediate customer gratification with the ultimate outcome of improving the patient’s quality-of-life perceptions”. |  |
| 51 | P. Klaus (Klaus, 2018) | 2018 | Healthcare (Luxury patients) | “The customer experience as "luxury patient experience" is the patient’s perception of psychologically and emotionally gratifying experiences, creating a constant mindset of receptiveness of luxury spending across the experience continuum with emphasis on experiencing wealth hospitality and embedded post-treatment care, ultimately enhancing their quality of life and wellbeing by focusing on their aspiration of substantiating and/or increasing their standing in society”. |  |
| 52 | V. Rusu, C. Rusu, F. Botella, D. Quiñones (Rusu *et al.*, 2018) | 2018 | General | “Customer experience (CX) is traditionally related to the Service Science field. It does not limit to the user – (software) product interaction but refers to the whole customer – company (or companies) interaction, through several products. When some of these products are software systems, the HCI interest in CX becomes obvious”. |  |
| 53 | H. Lee, K. K. Lee, and J. Choi (Lee *et al.*, 2018) | 2018 | General | “Customer experience (CX) represents interactivity between a customer and a service/product that provides sensory, emotional, cognitive, behavioral, and relational values to the customer; its ultimate goal is to build a holistic experience”. “CX is as a subset of the union of experiences; like user experience (UX), aims to build a holistic experience. The beginning of this experience is considered the moment at which the values that the CX provides are met. The holistic experience can be measured as the sum of values. In other words, it consists of a construct that can mediate the relation between the parts of UX and those of BX”. |  |
| 54 | R. N. Bolton, J. R. McColl-Kennedy, L. Cheung, A. Gallan, C. Orsingher, L. Witell, and M. Zaki (Bolton *et al.*, 2018) | 2018 | General | “Customer experience is defined as encompassing customers’ cognitive, emotional, social, sensory and value responses to the organization’s offerings over time, including pre- and post-consumption”. |  |
| 55 | F. Ali, W. G. Kim, J. Li, and H. Jeon (Ali *et al.*, 2018) | 2018 | Theme park | “Customer experience is the internal response to any direct or indirect contact with the theme park and its resources. It is a multi-dimensional and diverse construct, developed by various elements including: (i) the physical environment, (ii) interactions with staff, and (iii) interactions with other customers within the theme park”. |  |
| 56 | I. Belabbes and M. Oubrich (Belabbes and Oubrich, 2018) | 2018 | Telecommunica-tion (Mobile telecoms industry) | “Customer experience is the consequence of the physical and the emotional contact that the customer has with the company, its offers, or surroundings through the customer journey. It results in feelings, attitudes, and behaviors that the customer expresses in the form of satisfaction, loyalty, recommendation, and purchase”. |  |
| 57 | I. Benzarti and H. Mili (Benzarti and Mili, 2018) | 2018 | Management | “Customer experience is the set of interactions between a customer and a product or a company that involve a customer reaction which represents the degree of satisfaction of the customer towards the provided service”. |  |
| 58 | N. Behare, S. Waghulkar, S. A. Shah (Behare *et al.*, 2018) | 2018 | Business (Digital business strategy) | “Digital customer experience comprises of those experiences which were experienced through a digital interface, like a Laptop/computer, tablet, or smartphone. Hunting a product online by using a mobile app or through companies’ website, to find required information of any store at the nearest location, searching for tech support information on a smartphone, booking travel tickets, movie/concert/event’s ticket online etc. falls under digital customer experiences”. |  |
| 59 | H. Komulainen and H. Makkonen (Komulainen and Makkonen, 2018) | 2018 | Bank (Omnichannel banking) | “Customer experience is understood as an individual’s internal and subjective perception and a socially constructed and highly context-dependent entity that is susceptible to change and develop in terms of time”. |  |
| 60 | G. Beaudon and E. Soulier (Beaudon and Soulier, 2019) | 2019 | Market (Insurance) | “Customer Experience defines all interactions lived by a person and fostered by a brand from the customers’ point of views. These could be before, at the moment, or after the interaction”. |  |
| 61 | P. Signori, I. Gozzo, D. J. Flint, T. Milfeld, and B. Satinover Nichols (Signori *et al.*, 2019) | 2019 | Sustainability | “Sustainable Customer Experience (SCE) is a process that creates holistic value thanks to the customer’s engagement derived by sensations, feelings, cognitions, and behaviors evoked by sustainable stimuli, based on economic, social, and environmental sustainability”. |  |
| 62 | X. Dou, X. Zhu, J. Q. Zhang, and J. Wang (Dou *et al.*, 2019) | 2019 | Education (Entrepreneur-ship education) | “Customer experience is defined as a customer/student's journey with a service provider over his/her entire journey. That is, the totality of a customer's experience with an education program across multiple touchpoints”. |  |
| 63 | T. Fernandes and T. Pinto (Fernandes and Pinto, 2019) | 2019 | Bank (Retail banking) | “Customer experience is defined in retail banking as a multidimensional higher-order construct, corresponding to a four-factor structure consisting of Environment, Frontline Personnel, Moments-of-Truth, and Product Offerings”. |  |
| 64 | A. Bleier, C. M. Harmeling, and R. W. Palmatier (Bleier *et al.*, 2019) | 2019 | Retail (Online retailing) | “Customer experience comprises a customer’s subjective, multidimensional psychological response to a product’s presentation online”. |  |
| 65 | I. Anninoua and G. R. Foxall (Anninou and Foxall, 2019) | 2019 | Retail | “Customer experience is the totality of reinforcement and aversive consequences of the retail situation that consumers encounter over time within the context of interactions with retailers”. |  |
| 66 | M. Waqas, Z. L. Binti Hamzah, and N. A. Mohd Salleh (Waqas *et al.*, 2020) | 2020 | General | “The customer experience is a cognitive and affective state which arises from co-created cultural meanings given to a set of interactions between a customer and an object”. |  |
| 67 | L. Becker and E. Jaakkola (Becker and Jaakkola, 2020) | 2020 | General | “Customer experience comprises customers’ nondeliberate, spontaneous responses and reactions to offering-related stimuli along the customer journey. Customer experience ranges from ordinary to extraordinary representing the intensity of customer responses to stimuli”. “Customer experience stimuli reside within and outside firm-controlled touchpoints and can be viewed from multiple levels of aggregation”. “Customer experience stimuli and their interconnections affect customer experience in a dynamic manner. Customer experience is subjective and context specific, because responses to offering-related stimuli and their evaluative outcomes depend on customer, situational, and sociocultural contingencies”. | (Gonçalves *et al.*, 2020) |
| 68 | I. Khan, L. D. Hollebeek, M. Fatma, J. U. Islam, and I. Riivits-Arkonsuo (Khan *et al.*, 2020) | 2020 | Retail | “Customer experience is defined as a multidimensional construct that reflects the customer’s cognitive, emotional, behavioral, and sensorial responses to brand-related stimuli throughout their entire purchase journey”. |  |
| 69 | S. Pekovic and S. Rolland (Pekovic and Rolland, 2020) | 2020 | Retail (DIY retailing sector) | “Customer experience is defined as a multidimensional concept to integrate customer’s emotional, cognitive, sensorial, social, behavioral, and technological responses”. |  |
| 70 | W. Zhao and N. Deng (Zhao and Deng, 2020) | 2020 | Retail (Omnichannel retailing) | “Customer experience is the feeling of consumers in the shopping process affected by retailer’ services, shopping environment, product price and quality”. |  |
| 71 | A. De Keyser, K. Verleye, K. N. Lemon, T. L. Keiningham, and P. Klaus (De Keyser *et al.*, 2020) | 2020 | General | “Customer experience is formed through “touchpoints” which are embedded in a broader “context” and marked by a set of “qualities” that, together, result in a value judgment by the customer”. |  |
