# Supplementary material for: Understanding the customer experience in human-computer interaction: a systematic literature review
Source: PeerJ Comput Sci. 2023 Feb 10;9:e1219. doi: 10.7717/peerj-cs.1219 (PMC10280563; doi:10.7717/peerj-cs.1219)
Supplement: Supplemental Information 1 [file peerj-cs-09-1219-s001.zip › Supplementary material No2 - CX dimensions.docx]

## **Customer experience dimensions, components, or attributes.**

| **Nº** | **Author** | **Year** | **Domain** | **Number of dimen-sions** | **Name used to define the dimensions** | **Category name** | **Dimension name** | **Articles that cite this proposal** |
| --- | --- | --- | --- | --- | --- | --- | --- | --- |
| 1 | B. Schmitt (Schmitt, 1999) | 1999 | Marketing (Experiential marketing) | 5 | Module | - | Sense, Feel, Think, Act, Relate | (Srivastava and Kaul, 2016), (Hsu and Tsou, 2011), (Zhou and Mu, 2013), (Becker and Jaakkola, 2020), (Lemon and Verhoef, 2016), (Brakus *et al.*, 2009), (Chahal and Dutta, 2015), (Chen and Lin, 2015), (Liu *et al.*, 2017), (Klaus, 2015), (Walls *et al.*, 2011), (Hoyer *et al.*, 2020), (Yang and Wang, 2010), (Lyu *et al.*, 2019), (Zhao and Deng, 2020), (Tran *et al.*, 2017), (Pekovic and Rolland, 2020), (Roy *et al.*, 2020) |
| 2 | C. Shaw and J. Ivens (Shaw and Ivens, 2002) | 2002 | General | 11 | Category | - | Accessibility, Services, Quality, Activities, Environment, Products, Price, Range, Delivery, Location, Availability |  |
| 3 | C. Shaw and J. Ivens (Shaw and Ivens, 2002) | 2002 | Market (Utility market)  Retail | 5 | Element | - | Accessibility, Timeless, Accuracy, Reliability, Confidence |  |
| 4 | C. Shaw and J. Ivens (Shaw and Ivens, 2002) | 2002 | Market (Technical market) | 7 | Element | - | Accessibility, User friendliness, Accuracy, Reliability, Excitement, Innovation, Trust |  |
| 5 | B. J. Knutson, J. A. Beck, S. H. Kim, and J. Cha (Knutson and Beck, 2007) | 2007 | General | 7 | Dimension | - | Environment, Benefit, Accessibility, Convenience, Utility, Incentive, Trust | (Knutson *et al.*, 2009) |
| 6 | C. Gentile, N. Spiller, and G. Noci (Gentile *et al.*, 2007) | 2007 | General | 6 | Component | - | Sensorial, Emotional, Cognitive, Pragmatic, Lifestyle, Relational | (Belabbes and Oubrich, 2018), (“Phil” Klaus and Maklan, 2012), (Klaus *et al.*, 2013), (Liu *et al.*, 2017), (Klaus, 2015), (Hoyer *et al.*, 2020), (Tu and Zhang, 2013), (Salehi *et al.*, 2013), (Lyu *et al.*, 2019), (Pekovic and Rolland, 2020), (Rose *et al.*, 2012), (Belabbes *et al.*, 2017) |
| 7 | P. C. Verhoef, K. N. Lemon, A. Parasuraman, A. Roggeveen, M. Tsiros, and L. A. Schlesinger (Verhoef *et al.*, 2009) | 2009 | Retail | 6 | Determinant | - | Social environment, Service interface, Retail atmosphere, Assortment, Price, Promotions | (Belabbes and Oubrich, 2018), (Shin *et al.*, 2019), (Becker and Jaakkola, 2020), (Lemke *et al.*, 2011), (“Phil” Klaus and Maklan, 2012), (Klaus, 2013), (Klaus, 2015), (Pekovic and Rolland, 2020), (Singh, 2019) |
| 8 | J. J. Brakus, B. H. Schmitt, and L. Zarantonello (Brakus *et al.*, 2009) | 2009 | Brand (General brands) | 4 | Dimension | - | Sensory, Affective, Intellectual, Behavioral | (Srivastava and Kaul, 2016), (Lemon and Verhoef, 2016), (Hoyer *et al.*, 2020), (Zhao and Deng, 2020), (Anninou and Foxall, 2019), (Yoshida, 2017) |
| 9 | D. Grewal, M. Levy, and V. Kumar (Grewal *et al.*, 2009) | 2009 | Retail | 5 | Factor | - | Promotion, Price, Merchandise, Supply chain, Location | (Lemke *et al.*, 2011), (Kumar and Anjaly, 2017) |
| 10 | B. J. Knutson, J. A. Beck, S. H. Kim, and J. Cha (Knutson *et al.*, 2009) | 2009 | Hotel | 4 | Dimension | - | Benefit, Convenience, Incentive, Environment | (Ren *et al.*, 2016), (Lyu *et al.*, 2019) |
| 11 | S. H. Kim, J. Cha, B. J. Knutson, and J. A. Beck (Kim *et al.*, 2011) | 2011 | General | 7 | Dimension | - | Environment, Benefits, Convenience, Accessibility, Utility, Incentive, Trust |  |
| 12 | J. Yang and Y. Wang (Yang and Wang, 2010) | 2010 | Organization (Service climate) | 2 | Dimension | - | Customer perceptional experience, Customer emotional experience |  |
| 13 | H. Yu Hsu and H. Tsou (Hsu and Tsou, 2011) | 2011 | Blog (Online blog environments) | 5 | Dimension | - | Sense, Feel, Think, Act, Relate |  |
| 14 | P. Nambisan and J. H. Watt (Nambisan and Watt, 2011) | 2011 | Online product communities | 4 | Dimension | - | Pragmatic Experience, Hedonic Experience, Sociability Experience, Usability Experience |  |
| 15 | A. Walls, F. Okumus, Y. Wang, and D. J. Kwun (Walls *et al.*, 2011) | 2011 | Hotel (Luxury hotel) | 12 | Dimension | Physical environment | Ambience, Multisensory, Space and function, Signs, symbols, artifacts | (Ali *et al.*, 2018) |
|  |  |  |  |  |  | Human interaction | Employee, Fellow guest |  |
| 16 | P. Klaus and S. Maklan (Klaus and Maklan, 2011) | 2011 | Sport (Mountain-biking extreme sport camp) | 5 | Dimension | - | Hedonic enjoyment, Personal progression, Social interaction, Efficiency, Surreal feeling |  |
| 17 | J. Pareigis, B. Edvardsson, and B. Enquist (Pareigis *et al.*, 2011) | 2011 | Transport (Public transport) | 6 | Dimension | - | Customer processes, Other customers, Physical environment, Contact personnel, Provider processes, Outer environment | (Chauhan and Manhas, 2017) |
| 18 | P. Klaus (Klaus, 2011) | 2011 | Marketing | 4 | Dimension | - | Product experience, Outcome focus, Moments-of-truth, Peace-of-mind | (“Phil” Klaus and Maklan, 2012) |
| 19 | S. Maklan and P. Klaus (Maklan and Klaus, 2011) | 2011 | Bank (Retail banking) | 4 | Dimension | - | Product experience, Outcome focus, Moments-of-truth, Peace-of-mind |  |
| 20 | P. Klaus and S. Maklan (“Phil” Klaus and Maklan, 2012) | 2012 | Bank (Retail banking) | 4 | Dimension | - | Product experience, Outcome focus, Moments-of-truth, Peace-of-mind | (Fernandes and Pinto, 2019), (Klaus and Maklan, 2013), (Kumar and Anjaly, 2017) |
| 21 | P. Klaus and S. Maklan (Klaus and Maklan, 2013) | 2013 | Bank (Retail banking)  Mortgage  Fuel and service station  Retail (Luxury retailing) | 4 | Dimension | - | Product experience, Outcome focus, Moments-of-truth, Peace-of-mind |  |
| 22 | S. Rose, M. Clark, P. Samouel, and N. Hair (Rose *et al.*, 2012) | 2012 | Retail (Online retailing) | 2 | Component | - | Cognitive Experiential State, Affective Experiential State | (Singh, 2019) |
| 23 | R. Garg, Z. Rahman, M.N. Qureshi, and I. Kumar (Garg *et al.*, 2012) | 2012 | Bank (Banking sector) | 14 | Factor | Sensory | Servicescape, Online aesthetics | (Loureiro and Sarmento, 2017) |
|  |  |  |  |  |  | Affective | Customization, Core service, Online hedonic elements, Value addition |  |
|  |  |  |  |  |  | Cognitive | Convenience, Marketing mix, Online functional elements |  |
|  |  |  |  |  |  | Behavioral | Employees, Speed, Service process |  |
|  |  |  |  |  |  | Relational | Customer interaction, Presence of other customers |  |
| 24 | C. Zhang, Z. Wen-An, C. Jian, and G. Hai-Sheng (Chen *et al.*, 2012) | 2012 | Telecommunication (Telecommunication services) | 7 | Dimension | - | Reliability, Security/Privacy, Accessibility, Empathy, Tangibles, Responsiveness, Assurance |  |
| 25 | I. A. Wong and J. S. Wu (Wong and Wu, 2013) | 2013 | Casino | 15 | Attribute | Service environment | Atmosphere, Interior décor, Architecture, Facility |  |
|  |  |  |  |  |  | Employee service | Employee service quality, Employee appearance |  |
|  |  |  |  |  |  | Value | Access convenience, Price, Complementary offering |  |
|  |  |  |  |  |  | Hedonic and novelty | Hedonic service, Novelty and uniqueness |  |
|  |  |  |  |  |  | Brand experience | Brand image, Brand awareness, Brand attachment |  |
|  |  |  |  |  |  | Perceived luck | - |  |
| 26 | M. Salehi, M. Salimi, and A. Haque (Salehi *et al.*, 2013) | 2013 | Online product communities | 4 | Dimension | - | Pragmatic Experience, Sociability Experience, Usability Experience, Hedonic Experience |  |
| 27 | A. Rageh, T.C. Melewar, and A. Woodside (Rageh *et al.*, 2013) | 2013 | Hotel (Five star hotels) | 8 | Dimension | - | Comfort, Educational, Novelty, Recognition, Safety, Beauty, Relational, Hedonic |  |
| 28 | P. Klaus (Klaus, 2013) | 2013 | E-Commerce (Amazon) | 8 | Dimension | Functionality | Usability, Product presence, Communication, Social presence, Interactivity | (Pandey and Chawla, 2018) |
|  |  |  |  |  |  | Psychological Factors | Context familiarity, Trust, Value for money |  |
| 29 | Y. Q. Zhou and D. W. L. Mu (Zhou and Mu, 2013) | 2013 | Hotel (Tibetan cultural theme hotel) | 3 | Factor | - | Sense and feel experience, Act and relate experience, Think experience |  |
| 30 | J. Tu and M. Zhang (Tu and Zhang, 2013) | 2013 | Non-trading virtual community | 2 | Dimension | - | Emotional experience, Relationship experience |  |
| 31 | S. Bagdare and R. Jain (Bagdare and Jain, 2013) | 2013 | Retail | 4 | Dimension | - | Joy, Mood, Leisure, Distinctive |  |
| 32 | A. R. Walls (Walls, 2013) | 2013 | Hotel | 7 | Factor | Physical environment | Design, Physiological, Upkeep | (Ren *et al.*, 2016), (Lyu *et al.*, 2019) |
|  |  |  |  |  |  | Human interaction | Caring/attentive, Professionalism, Reliability, Guest-to-guest |  |
| 33 | P. Klaus, M. Gorgoglione, D. Buonamassa, U. Panniello, and B. Nguyen (Klaus *et al.*, 2013) | 2013 | Bank (Retail banking) | 3 | Dimension | - | Brand experience, Service (provider) experience, Post purchase experience | (Fernandes and Pinto, 2019) |
| 34 | S. Joshi (Joshi, 2014) | 2014 | Telecommunication (Cellular mobile devices) | 20 | Factor | Service performance/ delivery | Consistent service delivery, Appropriate charges, Value for money, Pricing better than competitor, Data connectivity and speed, Product better than competitor, Network coverage, Quality of service |  |
|  |  |  |  |  |  | Culture of customer care | Efficient customer care, Feel valued as customer, Company responsiveness, Complaint handling |  |
|  |  |  |  |  |  | Communication | Update of current/new services, Service matching customer needs, New technology launch/ time to market |  |
|  |  |  |  |  |  | Brand | Marketing communication, Brand image/reputation, Incentives/promotions by CSP |  |
|  |  |  |  |  |  | Environmental | Ease of recharge, Billing transparency, NDNC registration |  |
| 35 | F. Alia, K. Hussain, and N. A. Ragavan (Ali *et al.*, 2014) | 2014 | Hotel (Resort hotel) | 4 | Dimension | - | Entertainment, Esthetics, Education, Escapism |  |
| 36 | M. Sharma and D.S. Chaubey (Sharma and Chaubey, 2014) | 2014 | Bank (Banking sector) | 7 | Element | - | Positive outlook, Convenience, Responsiveness, Technological support, Ambiance, Marketing support services, Professionalism | (Fernandes and Pinto, 2019) |
| 37 | H. Zhang, Y. Lu, S. Gupta, and L. Zhao (Zhang *et al.*, 2014) | 2014 | Social commerce | 4 | Factor | - | Social presence, Flow, Informational support, Emotional support |  |
| 38 | H. Chahal and K. Dutta (Chahal and Dutta, 2015) | 2015 | Bank (Banking sector) | 3 | Dimension | Core experience | Cognitive, Affective, Behavioral | (Loureiro and Sarmento, 2017) |
|  |  |  |  |  |  | Relational experience,  Sensory experience | - |  |
| 39 | S. Peltola, H. Vainio, and M. Nieminen (Peltola *et al.*, 2015) | 2015 | Retail (Omnichannel retailing) | 4 | Factor | - | Organization and culture, Product information and pricing, Systems and logistics, Customer communication |  |
| 40 | D. Menachem, S. Joshi, S. Bhatia, A. Roy, and J. Saini (Menachem *et al.*, 2015) | 2015 | Telecommunication (Telecom operators) | 6 | Determinant | - | Brand image, Service, Delivery Experience, Network Experience, Customer Care Experience, Billing Experience, Store/Gallery and Web, Self Service, Experience | (Belabbes and Oubrich, 2018) |
| 41 | A.S. Sathish and P. Ganesan (Sathish and Ganesan, 2015) | 2015 | Retail | 8 | Dimension | - | Product value, Product variety, Internal store environment, Staff, Complaint handling, Store appearance, Store location, Store operations |  |
| 42 | Y. Pei, W. Xue, Y. Su, and D. Li (Pei, Xue, Su, *et al.*, 2015) | 2015 | E-Commerce (B2C e-commerce enterprises) | 3 | Factor | - | Website characteristics, Customer characteristics, Internet word of mouth |  |
| 43 | Y. Pei, W. Xue, D. Li, J. Chang, and Y. Su (Pei, Xue, Li, *et al.*, 2015) | 2015 | E-Commerce (B2C e-commerce enterprises) | 5 | Factor | - | Website usefulness, Website ease of use, Transaction costs, Internet word of mouth, Customer participation and interaction |  |
| 44 | P. Klaus (Klaus, 2015) | 2015 | General | 3 | Dimension | - | Brand experience, Service (firm) experience, Post-purchase/consumption experience |  |
| 45 | A. De Keyser, K. N. Lemon, P. Klaus, and T. L. Keiningham (De Keyser *et al.*, 2015) | 2015 | General | 5 | Element | - | Cognitive, Emotional, Physical, Sensorial, Social |  |
| 46 | K. N. Lemon and P. C. Verhoef (Lemon and Verhoef, 2016) | 2016 | Marketing | 5 | Construct | - | Cognitive, Emotional, Behavioral, Sensorial, Social | (Becker and Jaakkola, 2020) |
| 47 | L. Ren, H. Qiu, P. Wang, and P. M.C. Lin (Ren *et al.*, 2016) | 2016 | Hotel (Budget hotel) | 4 | Dimension | - | Tangible-sensorial experience, Staff relational/ interactional experience, Aesthetic perception, Location |  |
| 48 | M. Srivastava and D. Kaul (Srivastava and Kaul, 2016) | 2016 | Retail | 4 | Dimension | - | Feel, Relate, Sense, Think |  |
| 49 | S. Parise, P. J. Guinan, and R. Kafka (Parise *et al.*, 2016) | 2016 | Digital technology | 4 | Factor | - | Flow, Immersion, Cognitive fit, Emotional fit |  |
| 50 | J. Sujata, B. Sanjay, R. Arindom, and K. Madhurendra (Sujata *et al.*, 2016) | 2016 | Telecommunication (Cellular services) | 6 | Determinant | - | Brand Image, Service delivery, Network, Customer Care, Billing Experience, Store/ Gallery Experience |  |
| 51 | P. Deshwal and P. Bhuyan (Deshwal and Bhuyan, 2018) | 2016 | Healthcare (Cancer patient) | 5 | Construct (dimension) | - | Behavior experience, Comfort experience, Emotive experience, Environment experience, Social experience |  |
| 52 | S. M. Correia Loureiro and E. Moraes Sarmento (Loureiro and Sarmento, 2017) | 2017 | Bank (Online banking sector) | 6 | Attribute | - | Accessibility, Ease of doing business, Executional excellence, Personalized offering, Staff engagement, Value for money |  |
| 53 | N. T. Thi Tuyet and Y. Hara (Tran *et al.*, 2017) | 2017 | Retail (Traditional (wet market) and modern (supermarket)) | 4 | Factor | - | Sense, Perception, Feel, Relate |  |
| 54 | L. Wang, H. Tang, D. Liu, and C. Xing (Wang *et al.*, 2017) | 2017 | Brand (MI brand) | 3 | Dimension | - | Function Experience, Purchasing and Service Experience, Brand Experience |  |
| 55 | W. Liu, B. Sparks, and A. Coghlan (Liu *et al.*, 2017) | 2017 | Food and wine event | 4 | Dimension | - | Sensory, Fun, Discovery, Inspiration |  |
| 56 | V. Chauhan and D. Manhas (Chauhan and Manhas, 2017) | 2017 | Airline (Civil aviation sector) | 5 | Dimension | - | Hedonism, Novelty, Safety, Recognition, Comfort |  |
| 57 | I. Belabbes, A. Amine, and M. Oubrich (Belabbes *et al.*, 2017) | 2017 | Telecommunication (Mobile service market) | 5 | Determinant | Attrition and Retention | Loyalty, Churn, Boycott |  |
|  |  |  |  |  |  | Perception | Perceived quality, Negative perceived value, Positive perceived value |  |
|  |  |  |  |  |  | Affect | Positive Opinion, Negative Opinion, Feeling, User’s preference |  |
|  |  |  |  |  |  | Previous Experience and Attitude | Great experience, Bad experience, Choice assessment |  |
|  |  |  |  |  |  | QoS | Services usability, Technical Offer, Operator interaction |  |
| 58 | A. Kumar and B. Anjaly (Kumar and Anjaly, 2017) | 2017 | Retail (Online retailing (post-purchase)) | 6 | Dimension | - | Delivery, Product-In-Hand, Return and exchange, Customer support, Benefits, Feel-good factors |  |
| 59 | S. K. Roy, M. S. Balaji, S. Sadeque, B. Nguyen, and T. C. Melewar (Roy *et al.*, 2017) | 2017 | Retail (Smart retail technology) | 5 | Element | - | Relative advantage, Perceived enjoyment, Perceived control, Personalization, Interactivity |  |
| 60 | C. Khin-Whai Nicholas and A. Siew-Hoong Lee (Nicholas and Lee, 2017) | 2017 | Hotel | 3 | Factor | Satisfaction | Cleanliness, Overall environment, Value for money |  |
| 61 | F. Ali, W. Gon Kim, J. Li, and H. Jeon (Ali *et al.*, 2018) | 2018 | Theme park | 3 | Dimension | - | Physical environment, Interactions with staff, Interactions with other customers |  |
| 62 | I. Belabbes and M. Oubrich (Belabbes and Oubrich, 2018) | 2018 | Telecommunication (Mobile telecoms industry) | 5 | Dimension | - | Brand image, Pricing, Proximity and Customer care, Promotion, Usage tracking |  |
| 63 | S. Pandey and D. Chawla (Pandey and Chawla, 2018) | 2018 | Retail (Clothing e-retail) | 10 | Dimension | Functionality Factors | Interactivity, Informativeness, Visual engagement, Navigation and search ease |  |
|  |  |  |  |  |  | Psychological Factors | E-distrust, E-negative beliefs, E-self inefficacy, E-logistic ease, E-convenience, E-enjoyment |  |
| 64 | P. Wasan (Wasan, 2018) | 2018 | Bank (Retail banking) | 6 | Dimension | Functional Clues | Customization, Convenience, Credibility |  |
|  |  |  |  |  |  | Mechanic Clues | Service context |  |
|  |  |  |  |  |  | Humanic Clues | Compassion, Competence |  |
| 65 | R. Singh (Singh, 2019) | 2019 | Retail (Online grocery) | 4 | Dimension | Frictionless customer experience | Service excellence, Customer return on investment |  |
|  |  |  |  |  |  | Pleasurable customer experience | Aesthetics, Playfulness |  |
| 66 | I. Maslov (Maslov, 2019) | 2019 | Mass-Catering service | 5 | Attribute | Food | Variety of food, Food quality and taste |  |
|  |  |  |  |  |  | Service, Place, Price | - |  |
| 67 | X. Su, W. Lin, A. Xu, L. Huang, J. Wu, S. Cai, L. Sun, and W. Xu (Su *et al.*, 2019) | 2019 | Fresh products APPs | 3 | Dimension | - | Perceived experience, Affective experience, Trust experience |  |
| 68 | A. Bleier, C. M. Harmeling, and R. W. Palmatier (Bleier *et al.*, 2019) | 2019 | Retail (Online retailing) | 4 | Dimension | - | Informativeness, Entertainment, Social presence, Sensory appeal |  |
| 69 | T. Fernandes and T. Pinto (Fernandes and Pinto, 2019) | 2019 | Bank (Retail banking) | 4 | Dimension | - | Environment, Frontline Personnel, Moments-of-Truth, Product Offerings |  |
| 70 | J. Lyu, M. Li, and R. Law (Lyu *et al.*, 2019) | 2019 | P2P Accommodation | 7 | Dimension | - | Physical utility, Sensorial experience, Core service, Guest-host relationship, Sense of security, Social interaction, Local touch |  |
| 71 | S. Shi, Y. Wang, X. Chen, and Q. Zhang (Shi *et al.*, 2020) | 2020 | Retail (Omnichannel retailing) | 5 | Dimension | - | Connectivity, Integration, Consistency, Flexibility, Personalization |  |
| 72 | L. Becker and E. Jaakkola (Becker and Jaakkola, 2020) | 2020 | General | 5 | Dimension | - | Cognitive response, Affective response, Physical response, Sensorial response, Social response |  |
| 73 | W. D. Hoyer, M. Kroschke, B. Schmitt, K. Kraume, and V. Shankar (Hoyer *et al.*, 2020) | 2020 | New technologies | 3 | Dimension | - | Cognitive value, Sensory/emotional value, Social value |  |
| 74 | S. Pekovic and S. Rolland (Pekovic and Rolland, 2020) | 2020 | Retail (DIY retailing sector) | 6 | Dimension | - | Emotional, Cognitive, Sensory, Social, Behavioral, Technological |  |
| 75 | S. Zaharia and M. Schmitz (Zaharia and Schmitz, 2020) | 2020 | Retail (Online retailing) | 5 | Attribute | - | Cognitive, Fulfillment-related, Affective, Social, Sensorial |  |
| 76 | J. W. Shin, J. Y. Cho, and B. G. Lee (Shin *et al.*, 2019) | 2020 | Bank (Digital and traditional banks) | 4 | Dimension | - | Usefulness, Convenience, Employee-customer engagement (ECE), Security |  |
| 77 | W. Zhao and N. Deng (Zhao and Deng, 2020) | 2020 | Retail (Omnichannel retailing) | 3 | Dimension | - | Functional experience, Emotional experience, Seamless experience |  |
| 78 | S. K. Roy, R. L. Gruner, and J. Guo (Roy *et al.*, 2020) | 2020 | Retail | 4 | Dimension | - | Cognitive, Emotional, Physical and Sensorial, Social |  |
| 79 | F. Saberian, M. Amirshahi, M. Ebrahimi, and A. Nazemi (Saberian *et al.*, 2020) | 2020 | Digital platforms | 2 | Dimension | - | Hedonic Experience, Cognitive Experience |  |
| 80 | A. Al-Fadly (Al-Fadly, 2020) | 2020 | Construction | 4 | Aspect | - | Reputation, Confidence, Information, Expertise |  |
| 81 | L. Gonçalves, L. Patricio, J. G. Teixeira, and N. V. Wunderlich (Gonçalves *et al.*, 2020) | 2020 | Smart service (Smart energy service setting) | 11 | Dimension | Smart service specific dimensions | Controllability, Visibility, Self-configuration, Sustainability, Autonomy |  |
|  |  |  |  |  |  | Relationship dimensions | Relationship with the service provider, Community support |  |
|  |  |  |  |  |  | Technology-enabled dimensions | Cost-benefit, Accessibility, Ease of use, Ease of learning |  |
